# Supplementary material for: Secreted MbovP0145 Promotes IL-8 Expression through Its Interactive β-Actin and MAPK Activation and Contributes to Neutrophil Migration
Source: Pathogens. 2021 Dec 15;10(12):1628. doi: 10.3390/pathogens10121628 (PMC8707762; doi:10.3390/pathogens10121628)
Supplement: Supplementary file 1 [file pathogens-10-01628-s001.zip › supplementary figure legends.pdf]

Figure S1. The growth curves and procaryotic protein expression. A. Growth curves of the parental strain (HB0801), mutant strain (T6.93), and complemented strain (CT6.93) in axenic PPLO medium. Growth of *M. bovis* at each time point was determined with a plating assay. B. Growth curves of *M. bovis* strains under co-cultivation with EBL cells. C. SDS-PAGE analysis of expression and purification of recombinant MbovP0145(rMbovP0145). M, molecular weight marker; lane 1: non-induced sample, lane 2: induced sample, lane 3: purified rMbovP0145 protein. D. SDS-PAGE analysis of expression and purification of GST and recombinant GST-0145 protein. M: molecular weight marker, lanes 1 and 4: non-induced sample, lanes 2 and 5: induced sample, lanes 3 and 6: purified GST protein and GST-0145 protein.

Figure S2. Expression of IL-8 mRNA in EBL cells stimulated with *M. bovis* and LPS *in vitro*. A. Relative mRNA levels of IL-8 measured after *M. bovis* stimulated in EBL cells. 1×10^5 EBL cells were incubated with *M. bovis* of different MOI (10, 100, 1000) for 24 h. B. Relative mRNA levels of IL-8 measured after LPS stimulated in EBL cells. 1×10^5 EBL cells were incubated with LPS of different concentrations (10 μg/ml, 20 μg/ml, 30 μg/ml) for 24 h. Expression of IL-8 was evaluated using real-time PCR and indicated as relative values compared with unstimulated cells. Data are expressed as mean ± SE (**** p<0.001).
