# Supplementary figures and images for: Secreted MbovP0145 Promotes IL-8 Expression through Its Interactive β-Actin and MAPK Activation and Contributes to Neutrophil Migration
Source: Pathogens. 2021 Dec 15;10(12):1628. doi: 10.3390/pathogens10121628 (PMC8707762; doi:10.3390/pathogens10121628)

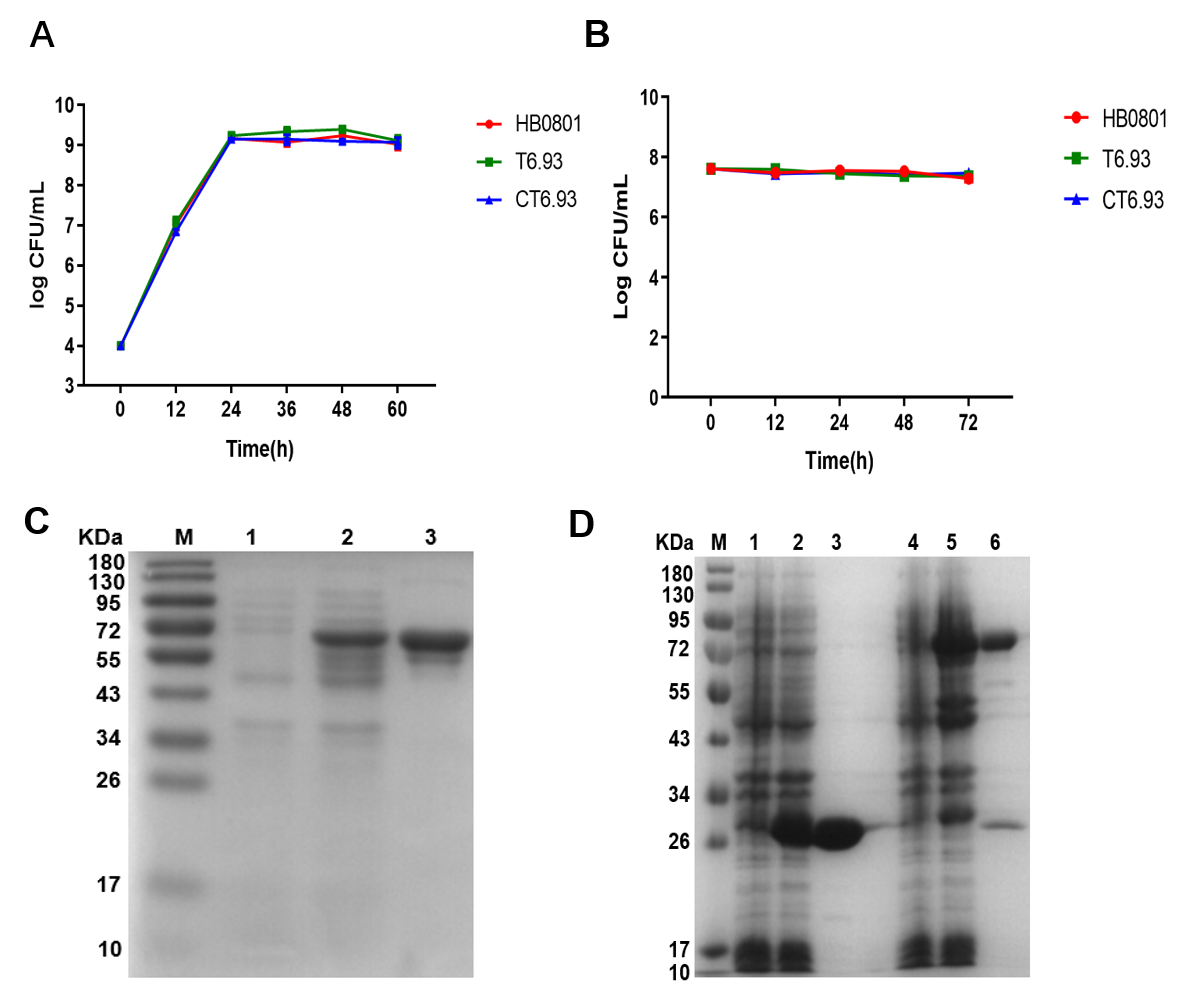

Supplement: Supplementary file 1 [file pathogens-10-01628-s001.zip › Fig S1.tif]

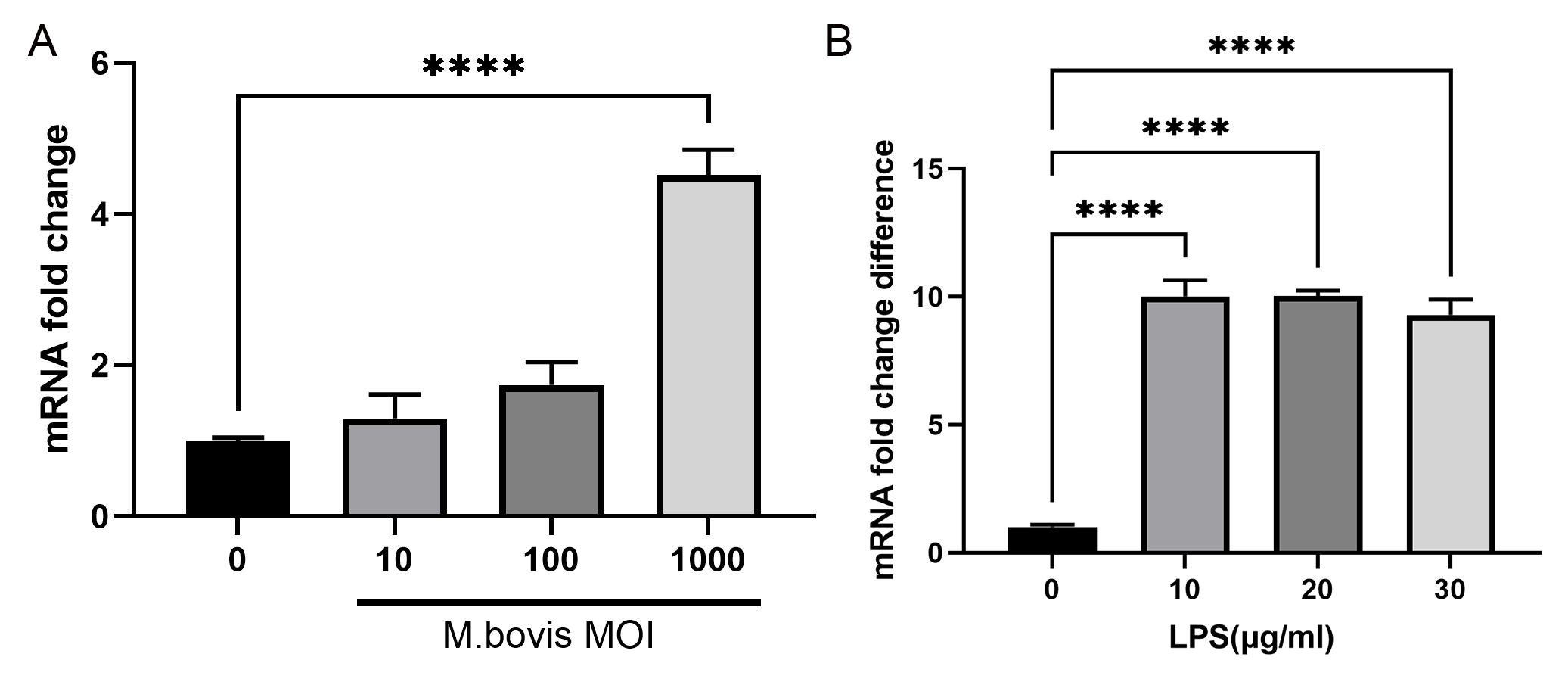

Supplement: Supplementary file 1 [file pathogens-10-01628-s001.zip › Fig S2.tif]
